# Dietary calcium intake and Renin Angiotensin System polymorphisms alter the blood pressure response to aerobic exercise: a randomized control design

**DOI:** 10.1186/1743-7075-4-1

**Published:** 2007-01-04

**Authors:** Linda S Pescatello, Debbie Turner, Nancy Rodriguez, Bruce E Blanchard, Gregory J Tsongalis, Carl M Maresh, Valerie Duffy, Paul D Thompson

**Affiliations:** 1 Department of Kinesiology, University of CT, Storrs, CT, USA; 2 Department of Allied Health Sciences, University of CT, Storrs, CT, USA; 3 Department of Nutritional Sciences, University of CT, Storrs, CT, USA; 4 Department of Pathology, Hartford Hospital, Hartford, CT, USA; 5 Department of Pathology, Dartmouth-Hitchcock Medical Center, Lebanon, NH, USA; 6 Division of Cardiology, Hartford Hospital, Hartford, CT, USA

## Abstract

**Background:**

Dietary calcium intake and the renin angiotensin system (RAS) regulate blood pressure (BP) by modulating calcium homeostasis. Despite similar BP regulatory effects, the influence of dietary calcium intake alone and combined with RAS polymorphisms on the BP response following acute aerobic exercise (i.e., postexercise hypotension) has not been studied. Thus, we examined the effect of dietary calcium intake and selected RAS polymorphisms on postexercise hypotension.

**Methods:**

Subjects were men (n = 50, 43.8 ± 1.3 yr) with high BP (145.3 ± 1.5/85.9 ± 1.1 mm Hg). They completed three experiments: non-exercise control and two cycle bouts at 40% and 60% of maximal oxygen consumption (VO_2_max). Subjects provided 3 d food records on five protocol-specific occasions. Dietary calcium intake was averaged and categorized as low (<880 mg/d = LowCa) or high (≥ 880 mg/d = HighCa). RAS polymorphisms (angiotensin converting enzyme insertion/deletion, ACE I/D; angiotensin II type 1 receptor, AT_1_R A/C) were analyzed with molecular methods. Genotypes were reduced from three to two: ACE II/ID and ACE DD; or AT_1_R AA and AT_1_R CC/AC. Repeated measure ANCOVA tested if BP differed among experiments, dietary calcium intake level and RAS polymorphisms.

**Results:**

Systolic BP (SBP) decreased 6 mm Hg after 40% and 60% VO_2_max compared to non-exercise control for 10 h with LowCa (p < 0.01), but not with HighCa (p ≥ 0.05). Under these conditions, diastolic BP (DBP) did not differ between dietary calcium intake levels (p ≥ 0.05). With LowCa, SBP decreased after 60% VO_2_max versus non-exercise control for 10 h among ACE II/ID (6 mm Hg) and AT_1_R AA (8 mm Hg); and by 8 mm Hg after 40% VO_2_max among ACE DD and AT_1_R CC/CA (p < 0.01). With HighCa, SBP (8 mm Hg) and DBP (4 mm Hg) decreased after 60% VO_2_max compared to non-exercise control for 10 h (p < 0.05), but not after 40% VO_2_max (p ≥ 0.05).

**Conclusion:**

SBP decreased after exercise compared to non-exercise control among men with low but not high dietary calcium intake. Dietary calcium intake interacted with the ACE I/D and AT_1_R A/C polymorphisms to further modulate postexercise hypotension. Interactions among dietary calcium intake, exercise intensity and RAS polymorphisms account for some of the variability in the BP response to exercise.

## Background

Recommendations to prevent and treat hypertension include weight loss, reduced sodium and adequate calcium intake, limited alcohol consumption, consumption of a diet rich in fruits, vegetables and low fat dairy products, and habitual physical activity [1]. The blood pressure (BP) lowering effects of dietary interventions are greater than those resulting from exercise [2,3]. Yet, the BP reductions resulting from combined nutrition and exercise lifestyle therapies are less than that expected based upon the estimated effects of either intervention used alone [3,4]. Variation in the individual BP response to lifestyle interventions as a result of interactions among environmental and genetic factors that are complex and not easily understood provides an explanation for these observations [5,6].

Epidemiological and experimental evidence indicate that calcium homeostasis has a role in BP regulation [8-13]. Low calcium dietary intake has been linked to higher BP and the development of hypertension, whereas calcium supplementation lowers BP. Ruidavets et al. [11] recently found significant and independent associations among higher dairy product and dietary calcium intakes and lower BP among 912 middle aged men [systolic BP (SBP) more so than diastolic BP (DBP)]. Dietary calcium intake is hypothesized to regulate BP by its influence on calcitrophic hormones, intracellular calcium concentration and vasculature reactivity [8-13].

The renin angiotensin system (RAS) is an important regulator of cardiovascular and renal function [14-16]. The RAS pathway begins with the production of renin that acts on angiotensinogen to form angiotensin I. Angiotensin converting enzyme (ACE) converts biologically inactive angiotensin I into angiotensin II, a potent vasopressor whose actions are mediated by the angiotensin II type 1 receptor (AT_1_R). These actions include mobilization of intracellular calcium, vasoconstriction, renal sodium reabsorption and aldosterone production. In population studies genetic variants of the RAS are associated with an increased risk of hypertension, notably the ACE insertion deletion (ACE I/D) and AT_1_R A/C polymorphisms [17,18].

Postexercise hypotension, or the immediate decrease in BP that occurs after a bout of aerobic exercise, is an accepted physiologic response to exercise with the largest BP decreases seen in those with the highest resting BP [19,20]. Yet, not all people with hypertension demonstrate postexercise hypotension for reasons that are not clear. We recently reported that the ACE I/D and AT_1_R A/C polymorphisms explain some of the variability in the BP response to acute aerobic exercise among men with elevated BP [21].

Dietary calcium intake [8-13] and the RAS [14-16] are important regulators of BP via their influence on calcium metabolism and vascular reactivity. Low dietary calcium intake paradoxically increases intracellular calcium concentration and is associated with high BP [8-13]. Angiotensin II regulates intracellular calcium concentration and peripheral vascular resistance by its actions on the AT_1_R [22,23]. Yet, the combined influence of dietary calcium intake and RAS polymorphisms associated with high BP on postexercise hypotension has not been studied.

The present investigation was designed to assess the effects of dietary calcium intake alone and in combination with the ACE I/D and AT_1_R A/C polymorphisms on postexercise hypotension among men with elevated BP. Since exercise induced BP reductions are greatest in those with the highest resting BP [19,20], we hypothesized that men consuming lower amounts of dietary calcium would experience greater BP reductions following a bout of aerobic exercise than men consuming higher amounts of dietary calcium. In addition, we postulated the exercise induced BP effects associated with dietary calcium intake would be further modulated by the ACE I/D and AT_1_R A/C polymorphisms. Consistent with our hypotheses, we found that interactions among dietary calcium intake, exercise intensity and RAS polymorphisms altered the BP response to acute aerobic exercise.

## Methods

### Subjects

Volunteers were 50 men between 18 and 55 yr with high normal to Stage 1 hypertension (SBP ≥ 130–159 and/or DBP ≥ 85–99 mm Hg). Subjects completed an informed consent approved by the Institutional Review Boards of the University of Connecticut and Hartford Hospital. If potential volunteers were taking medications or dietary supplements known to influence the BP response to exercise, they discontinued use for a least one month prior to the study. During this washout period, subjects taken off antihypertensive medications were monitored for evidence of accelerated hypertension. Men with excessive resting BP (SBP ≥ 160 and/or DBP ≥ 100 mm Hg) were excluded from further participation.

### Procedures

**Figure 1:**
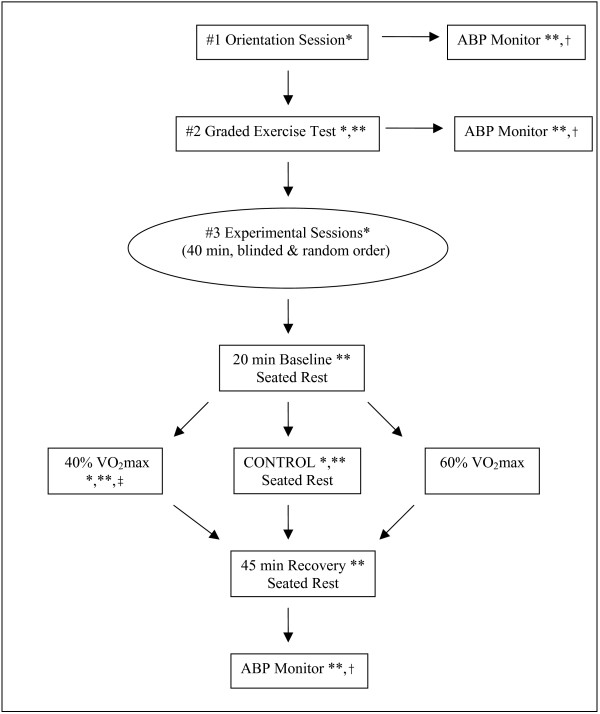
**Study design overview [20]**. ABP = ambulatory blood pressure; VO_2_max = maximal oxygen consumption; * 3 d dietary record beginning the morning of each testing session and continued for the next 2 d; ** Blood pressure taken throughout (see text for details); † worn until waking the next morning; ‡ includes 5 min warm-up and 5 min cool down periods to total 40 min of exercise.

The study design (Figure 1) and procedures have been described elsewhere [20,21]. Briefly, potential subjects completed an orientation session to familiarize them with the study, ensure their BP met the study inclusion criteria of high normal to Stage 1 hypertension, and educate them about the study procedures. In addition, waist circumference was measured, and height and weight were taken on a standard balance-beam scale (Model 339, Detecto, Webb City, MO) to calculate body mass index (kg/m^2^).

During the orientation session, participants were told to maintain their usual diet for the duration of the study. Prior to all testing sessions, subjects consumed a standard pre-testing meal of 1 c of low fiber cereal or a choice of one of the following: 2 slices of white toast, an English muffin or a 3.5 in diameter bagel. This meal was accompanied by 4 oz of 2%, 1% or skim milk, and 8 oz of orange juice. The entire meal was consumed 2 h prior to any testing session. Participants were also instructed to refrain from any caffeinated beverage the morning of all testing sessions and to drink caffeinated and alcoholic beverages in moderation (≤ 2 cups and drinks/d, respectively) throughout the study.

Participants received instruction on recording 3 d dietary records by the same registered dietitian on the mornings of the orientation session and graded cardiopulmonary exercise test. An example of an incomplete and a complete dietary record were shown to subjects in addition to verbal instruction on proper recording. Portion sizes were reviewed using food models as visuals. Subjects provided 3 d dietary records on five occasions (i.e., the orientation session, graded cardiopulmonary exercise test, and three experiments) (Figure 1). The registered dietitian performed a 24 h recall following the graded cardiopulmonary exercise test to validate 3 d dietary records over the same time period. Food records for 15 d were entered into the Nutrition Pro software program (N-Square Computing, Salem, OR) and analyzed by the registered dietitian for all subjects. Weight maintenance throughout the study was also used as an indication that volunteers were adhering to their usual dietary patterns. Men were weighed during the orientation session as well as prior to the graded cardiopulmonary exercise test and the three experiments to monitor weight maintenance.

At the completion of the orientation session, volunteers were attached to an ambulatory BP monitor (Accutracker II automatic noninvasive ambulatory BP monitor, Suntech Medical Instruments Inc., Raleigh, NC). The monitor was calibrated with a mercury sphygmomanometer until three successive measurements were within 5 mm Hg of values made by auscultation. The monitor was programmed to record BP approximately every 20 min. All subjects left the laboratory with instructions to proceed with their typical daily activities except for formal exercise and to return the monitor the following day. The computerized recordings were considered acceptable if at least 80% of the BP readings were obtained via the manufacturer's quality control criteria. If awake ambulatory BP averaged <135/85 mmHg, subjects were excluded from further participation [24].

Volunteers then completed a graded cardiopulmonary exercise test on a cycle ergometer (Monark Ergomedic 818E, Stockholm, Sweden) to determine the experimental exercise workloads. Maximal oxygen consumption (VO_2_max) was measured by breath-by-breath analysis of expired gases via an open circuit respiratory apparatus (Sensormedics Vmax 29 Metabolic Chart, SensorMedics Corp., Yorba Linda, CA). At the conclusion of the graded cardiopulmonary exercise test, volunteers were again attached to the ambulatory BP monitor to further acquaint them with the equipment.

Volunteers performed three 40 min experiments that were conducted in random order, performed at the same time of day, and were separated by a minimum of 48 h. The experiments included a non-exercise control session of seated rest, and two exercise bouts on a cycle ergometer performed at low (40% VO_2_max) and moderate intensity (60% VO_2_max) (Figure 1). All experiments began with a 20 min baseline period and were blinded to the subject until the conclusion of the baseline period. The exercise bouts consisted of 30 min of cycling at the designated exercise intensity with a 5 min warm up and 5 min cool down to total 40 min of exercise. Experiments then concluded with a 45 min recovery period of seated rest in the laboratory. During the experiments, heart rate was measured with a heart rate monitor (Model # 1902750, Polar Electro Inc, Woodbury, NY), and BP by auscultation by the same study investigator for every subject. Subjects left the laboratory wearing an ambulatory BP monitor until the next morning. The average hook up time to the ambulatory BP monitor was 12:30 pm.

### Genotype analysis

DNA was isolated from anticoagulated EDTA blood samples and typed for common RAS genetic variations. DNA was purified with molecular methods using the Puregene™ DNA Isolation Kit (Gentra systems, Inc. Minneapolis, MN). Separate primers that have been described elsewhere were used to detect RAS polymorphisms by the polymerase chain reaction and the restriction fragment length polymorphism method to determine mutations in the ACE I/D [25] and AT_1_R A/C [26] genes.

### Statistical analyses

A mean replacement strategy was used to replace the small number of missing BP values, and ambulatory BP values were then averaged at hourly intervals for purposes of statistical analyses. BP results are reported for 10 h after all experimental conditions because this is the time period over which subjects were awake and ambulating [20]. Descriptive statistics were generated on all study variables. Independent t-tests indicated that the 24 h food recall analysis following the graded cardiopulmonary exercise test did not differ from the analysis generated using the 3 d food records over this same time period (p ≥ 0.05). Because repeated measures analysis of variance revealed dietary calcium intake did not differ among the five monitoring periods (p ≥ 0.05), dietary calcium intake was averaged for 15 d and categorized by the sample median as LowCa (<880 mg/d, n = 25) or HighCa (≥ 880 mg/d, n = 25). The Chi-Square test was used to establish any deviation from the Hardy-Weinberg equilibrium among the RAS polymorphisms in the total sample and by dietary calcium intake level. Independent t-tests determined if physical characteristics and nutrient intake differed between dietary calcium intake level and RAS polymorphisms.

Repeated measures analysis of covariance tested if BP differed over time within and among experimental conditions (non-exercise control, 40% VO_2_max and 60% VO_2_max) and dietary calcium intake level (LowCa and HighCa). The ACE I/D (n = 49) and AT_1_R (n = 48) polymorphisms were distributed in accordance with the Hardy-Weinberg equilibrium having frequencies of ACE II 24%, ID 40% and DD 36% and AT_1_R AA of 56%, AC 40% and CC 4% in the total sample. Similarly, RAS polymorphisms were in Hardy-Weinberg equilibrium in the LowCa and HighCa groups. We previously found no significant differences in the BP responses among carriers of the I allele of the ACE I/D and the C allele of the AT_1_R A/C polymorphisms [21]. These genotypes were combined reducing the number of genotype classes in each RAS polymorphism from three to two (i.e., ACE II/ID and DD; and AT_1_R AA and CC/AC).

Repeated measures analysis of covariance then tested if BP differed over time within and among experimental conditions (non-exercise control, 40% VO_2_max and 60% VO_2_max), dietary calcium intake level (LowCa and HighCa), and the combined RAS genotype groups (ACE II/ID and DD; or AT_1_R AA and CC/AC). Covariates entered singularly in these analyses included average dietary intake of sodium, potassium, and magnesium; daily energy intake; % of total calcium derived from dairy sources; age; body mass index; and waist circumference. None of these covariates altered the primary BP outcomes found with dietary calcium intake level alone and with RAS polymorphisms so that unadjusted BP data are presented. All statistical analyses were performed with the Statistical Package for Social Sciences Base 14.0 (SPSS Inc., Chicago, IL) for Windows with p < 0.05 established as the level of significance.

## Results

### Subjects

Subjects (n = 50) were middle-aged, Caucasian men with elevated BP (Table 1). They were overweight and had below average physical fitness for men of their age [27]. Subjects with LowCa had higher awake ambulatory DBP and were older than those with HighCa (p < 0.05), while all other physical characteristics were not different between dietary calcium intake levels (p ≥ 0.05). In addition, physical characteristics were not different among the RAS polymorphisms (data not shown) (p ≥ 0.05).

**Table 1:** Mean physical characteristics (± SEM) of the study sample (n = 50) and by dietary calcium intake level.

**Characteristics**	**Total ** (n = 50)	**Low Calcium ** (n = 25)	**High Calcium ** (n = 25)
Age (yr)	43.8 ± 1.3	47.1 ± 1.2*	40.5 ± 2.3
Body mass index (kg/m^2^)	29.4 ± 0.7	28.6 ± 0.1	30.3 ± 1.2
Waist Circumference (cm)	101.9 ± 2.0	99.0 ± 2.4	104.2 ± 2.9
24 h Ambulatory SBP (mmHg)	141.4 ± 1.5	140.6 ± 2.2	142.2 ± 2.8
24 h Ambulatory DBP (mmHg)	83.3 ± 1.0	85.1 ± 1.5	81.5 ± 1.3
Ambulatory Awake SBP (mmHg)	145.3 ± 1.5	145.3 ± 2.4	145.3 ± 1.9
Ambulatory Awake DBP (mmHg)	85.9 ± 1.0	88.1 ± 1.6*	83.8 ± 1.3
Relative Maximum VO_2 _(ml·kg^-1^min^-1^)	31.3 ± 0.9	30.9 ± 1.1	31.7 ± 1.5

SBP = systolic blood pressure, DBP = diastolic blood pressure; VO_2 _= oxygen consumption

* p < 0.05 low (< median) versus high (≥ median) dietary calcium intake

### Dietary nutrient intake

Subjects consumed an average of 2577 ± 97 kcal/d of which 49% was from carbohydrates, 33% from total fat, 13% from saturated fat and 16% from protein (Table 2). Percent energy from saturated fat and average sodium intake exceeded current recommendations, and average magnesium intake was below recommended levels. All other nutrients were within recommended ranges [28]. Subjects generally consumed alcohol and caffeine (i.e., coffee) in moderate amounts of one to two glasses or cups per day, respectively. Men in the LowCa group consumed significantly less calcium, sodium, potassium, magnesium and daily calories than men in the HighCa group (p < 0.01) (Table 2). Dairy products provided 50% of total calcium intake. Subjects in the LowCa group had a lower percentage of calcium intake from dairy sources than men in the HighCa group, 45% versus 54%, respectively (p < 0.05). Other sources of calcium included daily multivitamins and fortified foods such as orange juice, cereal and enriched bread.

**Table 2:** Mean (± SEM) nutrient intake of study sample and by dietary calcium intake level.

**Nutrient**	**Total Sample **(n = 50)	**Low Calcium **(n = 25)	**High Calcium **(n = 25)
Calcium (mg/d)	1019.9 ± 76.2	659.4 ± 28.6‡	1380.4 ± 109.8
Energy Intake (kcal/d)	2576.8 ± 97.4	2224.0 ± 92.2‡	2929.5 ± 97.4
Carbohydrate (% kcal/d)	49.4 ± 1.0	48.5 ± 1.3	50.4 ± 1.5
Fat (% kcal/d)	33.2 ± 0.9	34.2 ± 1.3	32.2 ± 1.2
Saturated Fat (% kcal/d)	13.0 ± 0.4	12.9 ± 0.5	13.0 ± 0.7
Protein (% kcal/d)	15.5 ± 0.3	15.5 ± 0.5	15.5 ± 0.4
Sodium (mg)	4010.8 ± 395.0	3400.5 ± 183.8†	4621.0 ± 395.0
Potassium (mg)	2920.2 ± 126.8	2551.5 ± 111.0†	3288.9 ± 2.5.0
Magnesium (mg)	293.1 ± 15.3	249.1 ± 12.5†	337.2 ± 25.3
Alcohol (gm)	12.7 ± 2.4	12.1 ± 2.7	13.3 ± 3.9
Caffeine (mg)	141.6 ± 18.7	150.5 ± 22.3	132.7 ± 30.4

†p < 0.01, ‡p < 0.001 low (<median) versus high (≥ median) dietary calcium intake

### BP response

The BP findings following the experimental conditions (non-exercise control, 40% VO_2_max and 60% VO_2_max) in the total sample have been published [20]. They are briefly summarized for purposes of reference. SBP increased and DBP decreased after all experimental conditions compared to pre-experiment baseline values over 10 h (p < 0.001). However, SBP was reduced by 4.7 mm Hg after 60% VO_2_max and 2.4 mm Hg after 40% VO_2_max, and DBP was lower by 1.4 mm Hg after 40% VO_2_max only compared to non-exercise control over 10 h (p < 0.05).

### BP response by dietary calcium level

**Figure 2:**
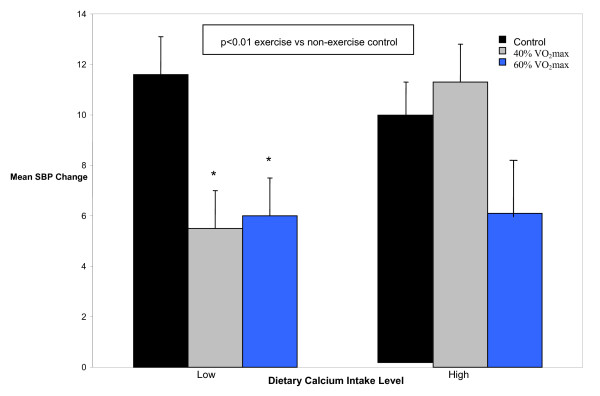
**Mean systolic blood pressure change (± SEM) from baseline after exercise and non-exercise control by dietary calcium intake level over 10 h**. SBP = systolic blood pressure; VO_2_max = maximal oxygen consumption; Low = < median dietary calcium intake; High = ≥ median dietary calcium intake.

**Figure 3:**
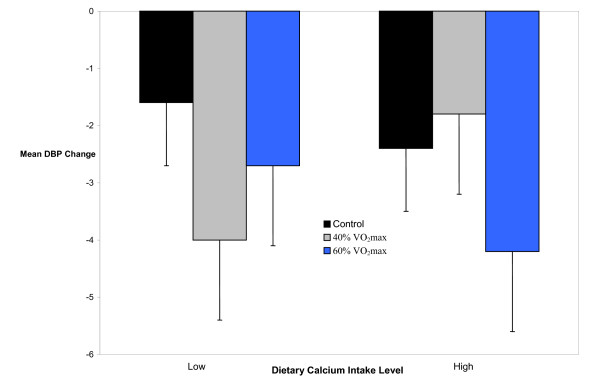
**Mean diastolic blood pressure change (± SEM) from baseline after exercise and non-exercise control by dietary calcium intake level for 10 h**. DBP = diastolic blood pressure; VO_2_max = maximal oxygen consumption; Low = < median dietary calcium intake; High = ≥ median dietary calcium intake.

SBP was reduced by approximately 6 mm Hg after exercise (40% and 60% VO_2_max) compared to non-exercise control for 10 h with LowCa (p < 0.01), but not HighCa (p ≥ 0.05) (Figure 2). Under these experimental conditions, DBP was not different between dietary calcium intake levels (p ≥ 0.05) (Figure 3).

We then determined whether the SBP reductions we found between exercise and non-exercise control with LowCa differed between dietary calcium levels for a given exercise condition (40% or 60% VO_2_max). The SBP response after 40% VO_2_max (a 6.0 mm Hg decrease versus a 1.4 mm Hg increase) compared to non-exercise control differed between LowCa and HighCa, respectively (p ≤ 0.05), but not after 60% VO_2_max (5.6 versus 3.7 mm Hg) (p ≥ 0.05).

### BP response by dietary calcium level and RAS polymorphisms

#### Low calcium dietary intake

##### ACE I/D polymorphism

Among carriers of the ACE I allele (i.e., ACE II/ID), SBP was reduced by 5.7 mm Hg from a pre-experiment baseline SBP of 126.6 ± 3.5 mm Hg after 60% VO_2_max compared to non-exercise control for 10 h (p < 0.01) (Table 3). Among ACE DD homozygotes, SBP tended to be lower by 9.8 mm Hg from a baseline SBP of 131.3 ± 5.6 mm Hg after 40% VO_2_max than non-exercise control for 10 h (p = 0.058). Under these conditions, DBP response was not different among the ACE I/D genotype groups (p ≥ 0.05).

**Table 3:** Mean blood pressure change (± SEM) from baseline after exercise and non-exercise control by ACE I/D genotype group over 10 h (95% confidence interval).

		Dietary	Calcium	Intake	Level	
	
	Low			High		
	
	Non-Exercise Control	40% VO_2_max	60% VO_2_max	Non-Exercise Control	40% VO_2_max	60% VO_2_max
		(n = 18)	**ACE**	**II/ID**	(n = 13)	

SBP	11.0 ± 2.0 (7.0,15.1)	7.5 ± 1.9 (3.8,11.3)	**5.3 ± 2.2† ** (0.9,9.7)	7.5 ± 2.4 (2.7,12.3)	13.8 ± 2.2 (9.4,18.2)	8.7 ± 2.6 (3.6,13.9)
DBP	-0.7 ± 1.5 (-3.9,2.4)	-3.5 ± 1.3 (-6.1,-0.9)	-2.6 ± 1.3 (-5.3,0.0)	-3.5 ± 1.8 (-7.2,0.1)	-1.2 ± 1.5 (-4.2,1.9)	-2.9 ± 1.5 (-6.0,0.2)

		(n = 7)	**ACE**	**DD**	(n = 11)	

SBP	10.3 ± 3.3 (3.1,16.8)	0.5 ± 3.0 (-5.5,6.5)	7.7 ± 3.5 (0.6,14.7)	10.0 ± 2.6 (4.8,15.2)	8.4 ± 2.4 (3.6,13.2)	2.5 ± 2.8* (-3.1,8.1)
DBP	-3.9 ± 2.5 (-8.9,1.1)	-5.2 ± 2.1 (-9.4,-1.0)	-3.0 ± 2.1 (-7.2,1.2)	-1.5 ± 2.0 (-5.5,2.5)	-3.0 ± 1.7 (-6.4,0.3)	-6.0 ± 1.7* (-9.3,-2.6)

ACE I/D = angiotensin converting enzyme insertion deletion polymorphism;

Low = < median dietary calcium intake; High = ≥ median dietary calcium intake;

VO_2_max = maximal oxygen consumption; SBP = systolic blood pressure; DBP = diastolic blood pressure.

*p < 0.05, **† **p < 0.01 exercise vs non-exercise control

We then examined whether the SBP reductions we found between exercise and non-exercise control within the ACE I/D genotype groups differed between genotype groups for a given exercise condition. The SBP reductions after 40% VO_2_max (3.5 versus 9.8 mm Hg) and 60% VO_2_max (5.7 versus 2.6 mm Hg) compared to non-exercise control differed between the ACE II/ID and DD genotype groups, respectively (p ≤ 0.05).

##### AT_1_R A/C polymorphism

Among AT_1_R AA homozygotes, SBP was reduced by 7.5 mm Hg from a pre-experiment baseline SBP of 127.8 ± 3.8 mm Hg after 60% VO_2_max compared to non-exercise control (p < 0.01) (Table 4). Among carriers of the AT_1_R C allele (i.e., AT_1_R AC/CC), SBP was lower by 7.5 mm Hg from a baseline SBP of 127.9 ± 5.0 mm Hg after 40% VO_2_max than non-exercise control for 10 h (p < 0.05). Under these conditions, DBP was not different among the AT_1_R A/C genotype groups (p ≥ 0.05). The SBP reductions after 60% VO_2_max (7.5 versus 2.1 mm Hg) compared to non-exercise control differed between the AT1R AA and CC/AC genotype groups (p ≤ 0.05), respectively; but not after 40% VO_2_max (5.2 versus 7.5 mm Hg) (p ≥ 0.05).

**Table 4:** Mean blood pressure change (± SEM) from baseline after exercise andnon-exercise control by AT_1_R A/C genotype group over 10 h (95% confidence interval).

		Dietary	Calcium	Intake	Level	
	
	Low			High		
	
	Non-Exercise Control	40% VO_2_max	60% VO_2_max	Non-Exercise Control	40% VO_2_max	60% VO_2_max
		(n = 16)	**AT_1_R**	**AA**	(n = 11)	

SBP	11.0 ± 2.2 (6.6,15.4)	5.8 ± 2.1 (1.5,10.1)	**3.5 ± 2.3† ** (-1.2,8.1)	13.8 ± 2.6 (8.5,19.1)	10.4 ± 2.6 (5.2,15.6)	6.9 ± 2.8* (1.2,12.5)
DBP	-1.9 ± 1.6 (-5.1,1.3)	-3.8 ± 1.4 (-6.6,-1.0)	-3.5 ± 1.4 (-6.4,-0.7)	0.9 ± 1.9 (-2.9,4.7)	-3.4 ± 1.7 (-6.8,0.0)	-3.1 ± 1.7 (-6.5,0.3)

		(n = 9)	**AT_1_R**	**CC/AC**	(n = 12)	

SBP	12.6 ± 2.9 (6.7,18.5)	5.1 ± 2.8* (-0.6,10.8)	10.5 ± 3.1 (4.2,16.7)	6.0 ± 2.5 (0.9,11.1)	11.8 ± 2.4 (6.8,16.7)	4.8 ± 2.7 (-0.6,10.2)
DBP	-1.2 ± 2.1 (-5.4,3.1)	-4.3 ± 1.9 (-8.0,-0.6)	-1.3 ± 1.9 (-5.0,2.5)	-5.2 ± 1.8 (-8.9,-1.5)	-0.6 ± 1.6 (-3.8,2.6)	-5.3 ± 1.6 (-8.6,-2.0)

AT_1_R A/C = angiotensin II type 1 receptor polymorphism; Low = < median dietary calcium intake; High = ≥ median dietary calcium intake; VO_2_max = maximal oxygen consumption; SBP = systolic blood pressure; DBP = diastolic blood pressure.

*p < 0.05, **† **p < 0.01 exercise vs non-exercise control

#### High calcium dietary intake

##### ACE I/D polymorphism

Among carriers of the ACE I allele, SBP and DBP were not different after exercise and non-exercise control (p ≥ 0.05) (Table 3). Among ACE DD homozygotes, SBP was reduced by about 7.5 mm Hg from a baseline SBP of 125.1 ± 3.2 mm Hg and DBP was lower by 4.5 mm Hg from a baseline DBP of 88.9 ± 2.6 mm Hg after 60% VO_2_max compared to non-exercise control for 10 h (p < 0.05). The SBP response (a 1.2 mm Hg increase versus a 7.5 mm Hg decrease) after 60% VO_2_max compared to non-exercise control differed between the ACE II/ID and DD genotype groups, respectively (p < 0.05). Under these conditions, there was a tendency for the DBP reductions (0.6 versus 4.5 mm Hg) (p = 0.065) to be different between the ACE II/ID and DD genotype groups as well.

##### AT_1_R A/C polymorphism

Among AT_1_R AA homozygotes, SBP was reduced by 6.9 mm Hg from a baseline SBP of 121.1 ± 3.1 mm Hg after 60% VO_2_max than non-exercise control for 10 h (p < 0.05) (Table 4); whereas DBP was not (p ≥ 0.05). Among carriers of the AT_1_R C allele, SBP and DBP were not different after exercise than non-exercise control for 10 h (p ≥ 0.05). The SBP reductions (6.9 verus 1.2 mm Hg) after 60% VO_2_max compared to non-exercise control tended to be different between the AT1R AA and CC/AC genotype groups, respectively (p = 0.086).

## Discussion

We examined the influence of dietary calcium intake on the BP response to acute aerobic exercise among 50 Caucasian, middle aged and overweight men with high normal to Stage 1 hypertension. In addition, we sought to determine if the BP response following acute endurance exercise was altered by interactions among dietary calcium intake and two RAS polymorphisms associated with hypertension, i.e., ACE I/D and AT_1_R A/C, that we [21] and others [29] have shown alter postexercise hypotension.

The new findings from this investigation are as follows. SBP was reduced by 6 mm Hg after exercise (40% and 60% VO_2_max) compared to non-exercise control over 10 h with LowCa, but not with HighCa (Figure 2). However, only the SBP response after 40% VO_2_max differed between dietary calcium intake levels. Under these conditions, DBP did not differ between dietary calcium intake levels (Figure 3). The acute SBP lowering effects of aerobic exercise were further modulated by interactions among dietary calcium level, exercise intensity and RAS polymorphisms.

With LowCa, SBP was lower after 60% VO_2_max among those less predisposed to cardiovascular disease risk based upon RAS genotype, i.e. ACE I allele carriers and AT_1_R AA homozygotes; and after 40% VO_2_max among those more predisposed to cardiovascular disease risk based upon their RAS genotype, i.e., ACE DD homozygotes and carriers of the AT_1_R C allele [17,18]. With HighCa, BP was lowered after 60% VO_2_max among ACE DD and AT_1_R AA homozygotes, but not after 40% VO_2_max. Our findings with LowCa and RAS polymorphisms reinforce the notion that lower intensity, aerobic exercise (40% VO_2_max) equivalent in physical exertion to leisurely walking is safer and better tolerated by people with high BP than more vigorous intensity, aerobic exercise (≥ 60% VO_2_max) [30]. Thus, lower intensity, aerobic exercise should be prescribed for those with high BP who are at increased risk of cardiovascular disease due to their dietary habits [8,11,12] and genetic predisposition [17,18].

Dietary calcium intake [8-13] and the RAS [14-18,31] are important regulators of BP via their effects on calcium metabolism. High levels of intracellular calcium increase vascular smooth muscle tone, peripheral vascular resistance, and responsiveness to the sympathetic and RAS systems; actions which elevate BP [9-11,32]. Paradoxically it is low but not high dietary calcium intake that stimulates an increase in parathyroid hormone leading to calcium mobilization from bone, increased intestinal calcium absorption, decreased renal calcium excretion, increased intracellular calcium concentration, and subsequently high BP. We found that a session of aerobic exercise interacted favorably with low but not high dietary calcium intake to lower BP. A possible explanation for these findings is that a bout of aerobic exercise acted on the sodium/calcium exchanger in vascular smooth muscle cells to extrude calcium from the intracellular space, thereby acutely decreasing BP among men with low dietary calcium intake who theoretically would have had the higher intracellular calcium concentration [31,33,34].

Klar et al. [22] have shown that angiotensin II inhibits renin gene expression in the kidney, thereby increasing intracellular calcium concentration. In addition, angiotensin II is a powerful signaling molecule that modulates calcium flux in brain stem nuclei that regulate vascular resistance via the AT_1_R [23]. Aerobic exercise activates the RAS in an intensity dependent manner [21]. The mechanisms by which dietary calcium intake and RAS polymorphisms would interact with exercise intensity to modulate postexercise hypotension are not clear. Nonetheless, our findings suggest the phenotypic expression of BP resulting from interactions among dietary calcium intake, exercise intensity and RAS polymorphisms reside in the balance achieved among them regarding the sodium/calcium exchanger in vascular smooth muscle and the general state of reactivity that they impose on the vasculature [9,10,16,21,31-36].

The complexity of the relationships we found among dietary calcium intake and RAS polymorphisms on postexercise hypotension were unexpected. Due to their preliminary nature and the small number of men in each of the designated dietary calcium intake level and RAS genotype groups, our findings are hypothesis generating. Of note is that they are consistent with our previous work on postexercise hypotension documenting exercise intensity [20] and RAS polymorphisms [21] differentially modulated the BP response to acute aerobic exercise. Our work continues to provide insight into possible reasons why 25–30% of the people with hypertension do not lower their BP after exercise [19]; observations that can be partially attributed to the complex interactions among environmental (i.e., diet and exercise) and genetic modulators of BP [6,7].

Other dietary nutrients have been implicated in BP regulation including potassium, magnesium and sodium [1,10,13,31,35]. Accordingly, we analyzed our findings for the potentially strong confounding influences of these micronutrients as well as daily energy intake, body mass index, waist circumference and age. Inclusion of these covariates singly into the statistical models did not alter the associations among dietary calcium intake level, RAS polymorphisms and the BP response to acute aerobic exercise. The type of food source of calcium may also have influenced our results [11] so that we examined our data for % of total calcium derived from dairy sources and found no difference in our major BP outcomes.

This study was subject to several limitations. Dietary calcium intake was quantified from multiple 3 d food record sets in a free living population. However, the degree of interaction with the same registered dietitian throughout the study was intensive and assures a certain level of confidence in the dietary data. More importantly, others have documented that 3 d food records are sufficient to provide information regarding routine nutrient intake [37]. In addition, there were no differences noted in total energy, diet composition and micronutrient intake between the 24 h recall and 3 d food record nutrient analyses. The small number of subjects in some of the dietary calcium intake level and RAS genotype groups limit the conclusions that can be made about the interactions of diet and genetic variation on postexercise hypotension. Nonetheless, BP differed *within *a given dietary calcium level, exercise condition and RAS genotype group as well as *between *RAS genotype groups for a given dietary calcium level and exercise condition, suggesting the BP differences we found are partially due to genetic variation. Additionally, results from this study can be generalized only to the subjects enrolled-white, middle aged and overweight men with high normal to Stage 1 hypertension who are not taking medication for their high BP.

## Conclusion

The novel findings of this investigation were that the BP response to acute aerobic exercise was modulated by interactions among dietary calcium intake, exercise intensity and RAS polymorphisms. Specifically, with LowCa, SBP was reduced after 60% VO_2_max among those less predisposed to cardiovascular disease risk based upon their RAS genotype; and after 40% VO_2_max among those more predisposed to cardiovascular disease risk based upon their RAS genotype. With HighCa, BP was lower after 60% VO_2_max (but not 40% VO_2_max) among ACE DD and AT1R AA homozygotes. Interactions among dietary calcium intake, exercise intensity and RAS polymorphisms provide insight into why most people manifest postexercise hypotension but some do not. Further investigation is needed to validate our findings in a larger, more ethnically diverse sample of men and women in which dietary calcium intake is manipulated so that definitive conclusions can be made about the complex relationships we observed among dietary calcium intake, exercise intensity and RAS polymorphisms on the immediate BP lowering effects of aerobic exercise.

## Abbreviations

Angiotensin converting enzyme insertion deletion polymorphism ACE I/D

Angiotensin II type 1 receptor polymorphism AT_1_R A/C

Blood Pressure BP

Diastolic blood pressure DBP

HighCa Calcium intake ≥ 880 mg/d

LowCa Calcium intake < 880 mg/d

Maximal oxygen consumption VO_2 _max

Renin angiotensin system RAS

Systolic blood pressure SBP

## Authors' contributions

LSP: Concept and design; data acquisition; statistical expertise; data analysis and interpretation; and primary manuscript writer.

DT: Data acquisition; statistical expertise; data analysis and interpretation; and significant manuscript writer.

NR: Concept and design; data acquisition and interpretation; and manuscript writer and reviewer for important intellectual content.

BB: Data acquisition; statistical expertise; data analysis and interpretation; and manuscript reviewer.

GT: Performed genetic analyses; data interpretation; and manuscript reviewer for important intellectual content.

CM: Data acquisition; data interpretation; and manuscript reviewer for important intellectual content.

VD: Data acquisition and interpretation; and manuscript reviewer for important intellectual content.

PT: Data acquisition; data interpretation; and manuscript reviewer for important intellectual content.

## References

[B1] Chobanian AV, Bakris GL, Black HR, Cushman WC, Green LA, Izzo JL, Jones DW, Materson BJ, Oparil S, Wright JT, Roccella EJ (2003). Seventh Report of the Joint National Committee on Prevention, Detection, Evaluation, and Treatment of High Blood Pressure. JNC 7-Complete Version. Hypertension.

[B2] Seals DR, Tanaka H, Clevenger CM, Monahan KD, Reiling MJ, Hiatt WR, Davey KP, DeSouza CA (2001). Blood pressure reductions with exercise and sodium restriction in postmenopausal women with elevated systolic pressure: Role of arterial stiffness. J Am Coll Cardiol.

[B3] Shaw K, Gennat H, O'Rourke P, Del Mar C (2006). Exercise for overweight or obesity. Cochrane Database of systematic Reviews.

[B4] PREMIER Collaborative Research Group Writing Group (2003). Effects of comprehensive lifestyle modification on blood pressure control main results of the PREMIER clinical trial. JAMA.

[B5] Egan BM (2003). Reproducibility of BP responses to changes in dietary salt compelling evidence for universal sodium restriction. Hypertension.

[B6] Heck AL, Barroso CS, Callie ME, Bray MS (2004). Gene-nutrition interaction in human performance and exercise response. Nutri.

[B7] Saavedra JM (2005). Studies on genes and hypertension: a daunting task. J Hypertens.

[B8] Allender PS, Cutler JA, Follmann D, Cappuccio FP, Pryer J, Elliott P (1996). Dietary calcium and blood pressure a meta-analysis of randomized clinical trials. Ann Intern Med.

[B9] Jorde R, Svartberg J, Sundsfjord J (2005). Serum parathyroid hormone as a predictor of increase in systolic blood pressure in men. J Hypertens.

[B10] Resnick LM (1999). The role of dietary calcium in hypertension: a hierarchal overview. Am J Hypertens.

[B11] Ruidavets J-B, Bongard V, Simon C, Dallongeville J, Ducimetiere P, Arveiler D, Amouyel P, Bingham A, Ferrierres (2006). Independent contribution of dairy products and calcium intake to blood pressure variations at a population level. J Hypertens.

[B12] Van Mierlo LAJ, Arends LR, Streppel MT, Zeegers MPA, Kok FJ, Grobbee DE, Geleijnse JM (2006). Blood pressure response to calcium supplementation: a meta-analysis of randomized controlled trials. J Hum Hypertens.

[B13] Zemal M (2001). Calcium modulation of hypertension and obesity: mechanisms and implications. J Am Coll Nutr.

[B14] Der Sarkissian S, Huentelman MJ, Stewart J, Katovich MJ, Raizada MK (2006). Ace2: a novel therapeutic target for cardiovascular diseases. Prog Biophys Mol Biol.

[B15] Levy BI (2004). Can angiotensin II type 2 receptors have deleterious effects in cardiovascular disease? implications for therapeutic blockade of the rennin-angiotensin system. Circulation.

[B16] Laragh JH, Sealey JE (2003). Relevance of the plasma renin hormonal control system that regulates blood pressure and sodium balance for correctly treating hypertension and for evaluating ALLHAT. Am J Hypertens.

[B17] Henskens LH, Spiering W, Stoffers HE, Soomers FL, Vlietinck RF, de Leeuw PW, Kroon AA (2003). Effects of ACE I/D and AT1R-A1166C polymorphisms on blood pressure in a healthy normotensive primary care population: first results of the Hippocates study. J Hypertens.

[B18] Staessen JA, Wang JG, Brand E, Barlassina C, Birkenhager WH, Herrmann SM, Fagard R, Tizzoni L, Bianchi G (2001). Effects of three candidate genes on prevalence and incidence of hypertension in a Caucasian population. J Hypertens.

[B19] Pescatello LS, Franklin BA, Fagard R, Farquhar WB, Kelley GA, Ray CA (2004). American College of Sports Medicine position stand. Exercise and hypertension. Med Sci Sports Exerc.

[B20] Pescatello LS, Guidry MA, Blanchard BE, Kerr A, Taylor AL, Johnson AN, Maresh CM, Rodriguez N, Thompson PD (2004). Exercise intensity alters postexercise hypotension. J Hypertens.

[B21] Blanchard BE, Tsongalis GJ, Guidry MA, LaBelle LA, Poulin M, Taylor AL, Maresh CM, Devaney J, Thompson PD, Pescatello LS (2006). RAAS polymorphisms alter the acute blood pressure response to aerobic exercise among men with hypertension. Eur J Appl Physiol.

[B22] Klar J, Sigl M, Obermayer B, Schweda, Kramer BK, Kurtz A (2005). Calcium inhibits renin gene expression by transcriptional and posttranscriptional mechanisms. Hypertension.

[B23] Wang F, Anrather J, Glass MJ, Tarsitano MJ, Zhou P, Frys KA, Pickel VM, Iadecola C (2006). Nox2, CA^2+^, and protein kinase c play a role in angiotensin II-induced free radical production in nucleus tractus solitarius. Hypertension.

[B24] Pickering TG, Hall JE, Appel LJ, Falkner BE, Graves J, Hill MN, Jones DW, Kurtz T, Sheps SG, Roccella EJ (2005). Recommendations for blood pressure measurement in humans and experimental animals Part 1: Blood pressure measurement in humans A statement for professionals from the subcommittee of professional and public education of the American Heart Association Council on High Blood Pressure Research. Hypertension.

[B25] Shanmugam V, Sell KW, Saha BK (1993). Mistyping ACE heterozygotes. PCR Methods Appl.

[B26] Berge KE, Berg K (1998). Polymorphisms at the angiotensinogen (AGT) and angiotensin II type 1 receptor (AT1R) loci and normal blood pressure. Clin Genet.

[B27] (2006). American College of Sports Medicine. ACSM's Guidelines for Exercise Testing and Prescription.

[B28] (1997). Institute of Medicine Standing Committee on Scientific Evaluation of Dietary Reference Intakes. Food and Nutrition Board. Dietary reference intakes for calcium, phosphorus, magnesium, vitamin D, and fluoride.

[B29] Taylor-Tolbert NS, Dengel DR, Brown MD, McCole SD, Pratley RE, Ferrell RE, Hagberg JM (2000). Ambulatory blood pressure after acute exercise in older men with essential hypertension. Am J Hypertens.

[B30] Giri S, Thompson PD, Kiernan FJ, Clive J, Fram DB, Mitchel JF, Hirst JA, McKay RG, Waters DD (1999). Clinical and angiographic characteristics of exertion-related acute myocardial infarction. JAMA.

[B31] Siani A, Russo P, Cappuccio FP, Iacone R, Venezia A, Russo O, Barba G, Iacoviells L, Strazzullo P (2004). Combination of renin-angiotensin system polymorphisms is associated with altered renal sodium handling and hypertension. Hypertens.

[B32] Gennari G, Nami R, Gonnelli S (1995). Hypertension and primary hyperparathyroidism: the role of adrenergic and renin-angiotensin-aldosterone systems. Miner Electrolyte Metab.

[B33] Jolma P, Kalliovalkama J, Tolvanen J, Koobi P, Kahonen M, Hutri-Kanonen N, Wu X, Porsti I (2000). High-calcium diet enhances vasorelaxation in nitric oxide-deficient hypertension. Am J Physiol Heart Circ Physiol.

[B34] Schweda F, Seebauer H, Kremer BK, Kurtz A (2001). Functional role of sodium-calcium exchange in the regulation of renal vascular resistance. Am J Physiol Renal Physiol.

[B35] Akita S, Sacks FM, Svetkey LP, Conlin PR, Kimura G (2003). Effects of the dietary approaches to stop hypertension (DASH) diet on the pressure-natriuresis relationship. Hypertens.

[B36] Hayashi A, Kobayashi A, Takahashi R, Suzuki F, Nakagawa T, Kimotro K (2000). Effects of voluntary running exercise on blood pressure and renin angiotensin system in spontaneously hypertensive rats and normotensive Wistar-Kyoto rats. J Nutr Sci Vitaminol.

[B37] Thompson F, Subar A, Coulston AM, Rock CL, Monsen ER (2001). Dietary assessment methodology. Nutrition in the Prevention and Treatment of Disease.

